# Protective Effect of Caffeine and Chlorogenic Acids of Coffee in Liver Disease

**DOI:** 10.3390/foods13142280

**Published:** 2024-07-20

**Authors:** Daniela Di Pietrantonio, Valeria Pace Palitti, Angelo Cichelli, Stefania Tacconelli

**Affiliations:** 1 Department of Innovative Technologies in Medicine and Dentistry, “G. d’Annunzio” University, Via dei Vestini 31, 66100 Chieti, Italy; dipietrantonio@liceomasci.edu.it; 2 Internal Medicine and Hepatology Unit, Azienda Sanitaria Locale, Via R. Paolini 47, 65125 Pescara, Italy; valeria.pacepalitti@asl.pe.it; 3 Department of Neuroscience, Imaging and Clinical Science, “G. d’Annunzio” University, Via dei Vestini 31, 66100 Chieti, Italy

**Keywords:** caffeine, chlorogenic acid, liver disease, protective effect

## Abstract

Coffee is one of the most widely consumed beverages in the world due to its unique aroma and psychostimulant effects, mainly due to the presence of caffeine. In recent years, experimental evidence has shown that the moderate consumption of coffee (3/4 cups per day) is safe and beneficial to human health, revealing protective effects against numerous chronic metabolic diseases such as diabetes, cardiovascular, neurodegenerative, and hepatic diseases. This review focuses on two of coffee’s main bioactive compounds, i.e., caffeine and chlorogenic acids, and their effects on the progression of chronic liver diseases, demonstrating that regular coffee consumption correlates with a lower risk of the development and progression of non-alcoholic steatohepatitis, viral hepatitis, liver cirrhosis, and hepatocellular carcinoma. In particular, this review analyzes caffeine and chlorogenic acid from a pharmacological point of view and explores the molecular mechanism through which these compounds are responsible for the protective role of coffee. Both bioactive compounds, therefore, have antifibrotic effects on hepatic stellate cells and hepatocytes, induce a decrease in connective tissue growth factor, stimulate increased apoptosis with anti-cancer effects, and promote a major inhibition of focal adhesion kinase, actin, and protocollagen synthesis. In conclusion, coffee shows many beneficial effects, and experimental data in favor of coffee consumption in patients with liver diseases are encouraging, but further prospective studies are needed to demonstrate its preventive and therapeutic role in chronic liver diseases.

## 1. Introduction

**Figure 1:**
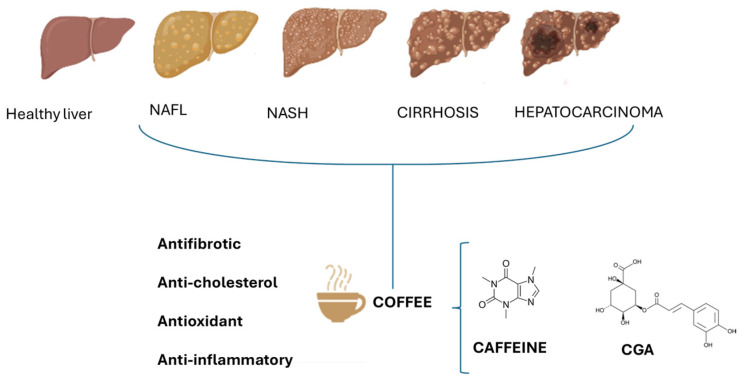
**Effect of caffeine and chlorogenic acids (CGAs) on different stages of NAFLD**. The beneficial effects of coffee are due to the potential antifibrotic, anti-cholesterol, antioxidant, and anti-inflammatory actions of caffeine and CGAs.

Coffee is one of the world’s most popular psychoactive beverages, and Italy ranks seventh in global consumption with around 95 million cups every day [1]. For a long time, coffee was considered detrimental to health [2], but epidemiological data and results from years of studies have provided opposite and encouraging results, showing that the risk of numerous chronic diseases was inversely correlated with regular, moderate coffee consumption [3]. Coffee is a mixture of complex compounds, with multifaceted health benefits, particularly for diseases such as cancer, type 2 diabetes mellitus, liver injury, liver cirrhosis, depression, and neurological and cardiovascular disorders [4,5,6]. Coffee is also used during physical activity because it increases the serum levels of catecholamines and induces performance improvement. Furthermore, coffee may promote an improvement in hepatic steatosis and fibrosis, reducing the risk of progression to cirrhosis and of developing hepatocellular carcinoma (HCC). Numerous substances are present in coffee, including some phenols such as chlorogenic acids; diterpenes such as cafestol and kahweol; some alkaloids such as caffeine and trigonelline; and many other bioactive compounds and their metabolites. Moderate coffee consumption (1–5 cups/daily) is considered beneficial in reducing the risk of all-cause mortality, heart failure, and cardiovascular diseases [3,7,8], whereas excessive coffee intake (>6 cups per day) seems to be associated with a slightly higher cardiovascular risk [9]. However, there is evidence in the literature that decaffeinated coffee may have benefits similar to regular coffee, albeit to a lesser extent, indicating that in addition to caffeine, other components may contribute to the health-protective effects. Both types of coffee showed preventive effects on chronic liver diseases, including fibrosis, cirrhosis, and HCC [9]. Although there have been numerous studies with conflicting conclusions about whether coffee consumption is beneficial or harmful to human health, coffee’s image as a superfunctional food has contributed to its increased consumption worldwide [7]. The purpose of this review is to give evidence based on the literature for the positive correlations between the use of coffee, in terms of caffeine and chlorogenic acids (CGAs), and multiple liver outcomes (Figure 1). Although other alkaloids, such as theophylline and theobromine, are also present in coffee, caffeine is found in much higher concentrations [10,11], accounting for 1.5–3% of the dry basis. CGAs, on the other hand, are the major phenolic compounds accounting for 6–12% of the dry basis. Importantly, even if variable in %, coffee represents the richest dietary source of CGAs [10].

## 2. Protective Role of Coffee in Liver Diseases

Liver diseases cause more than two million deaths per year (from cirrhosis, viral hepatitis, and liver neoplasia) and 4% of all the deaths worldwide (1 in 25 deaths) [12]. Hepatitis B virus (HBV) and hepatitis C virus (HCV), alcohol-related liver disease, steatotic liver disease [currently termed metabolic dysfunction-associated steatotic (MASLD)], and autoimmune liver disease are the main causes of chronic liver diseases. Globally, the prevalent etiology of liver cirrhosis is HBV infection in 42% and HCV infection in 21% of the cases [12].

Although liver cirrhosis of viral etiology remains the leading cause of liver-related mortality, followed by alcohol-related chronic liver disease, there is an exponential rise in steatosis- and metabolic-related chronic liver disease due to the epidemic increase in obesity and diabetes mellitus. Unfortunately, chronic liver disease is still often diagnosed in advanced stages when complications occur. Therefore, it will be crucial to direct resources towards primary prevention, early diagnosis, and increased access to treatment [13,14].

### 2.1. HBV/HCV

In 2020, more than one million deaths related to HBV and HCV infection were reported. HBV infection is, in most cases, asymptomatic. In adults, the disease becomes chronic in approximately 5–10% of the cases and in 20% of the cases, it can progress to cirrhosis within about 5 years [12]. HCC is another frequent complication of chronic hepatitis, especially in patients with cirrhosis. In contrast to hepatitis C, the risk of developing HCC in HBV carriers can occur without cirrhosis. HBV infection in high-endemic countries accounts for up to 90% of HCC [15].

A French study showed that the intake of three or more cups of coffee per day in chronic hepatitis B patients was associated with a reduced risk of liver fibrosis as measured by non-invasive biomarkers [16]. Furthermore, among chronic HBV carriers, patients drinking coffee ≥4 times per week had a 59% lower liver cancer risk compared to those who did not drink coffee [17]. Among HCV patients, up to 30% can spontaneously eliminate the virus within 6 months after an acute infection, while approximately 70% develop a chronic infection that is associated with a 15–30% risk of progression to cirrhosis within 20 years, and subsequent risk of developing serious complications including HCC. Coffee drinkers are more likely to respond to HCV pharmacological therapies [18] and may have a lower risk of progression to end-stage liver disease and HCC.

### 2.2. Hepatic Steatosis

Non-alcoholic fatty liver disease (NAFLD) or steatotic liver disease (SLD) is an emerging liver disease, highly associated with obesity and diabetes. NAFLD encompasses histological features that begin with lipid accumulation in the hepatocytes (simple steatosis) without significant lobular inflammation or liver fibrosis, with the possibility of progressing to non-alcoholic steatohepatitis (NASH), characterized by the balloniform degeneration of hepatocytes and diffuse lobular inflammation which may induce various stages of fibrosis. The progression towards fibrosis can lead to cirrhosis and increased cardiovascular risk, liver-related morbidity, and mortality. Cirrhosis may be complicated by HCC or liver failure. The risk of developing HCC is also relevant in non-cirrhotic NASH patients. Recently, the Food and Drug Administration (FDA) has approved the first treatment option (resmetirom) for adults with NASH with moderate to advanced liver fibrosis [19,20] in addition to lifestyle modification. In fact, significant weight loss (≥5%) is associated with a reduction in steatosis, while a weight loss of ≥7–10% has been shown to improve steatohepatitis and fibrosis. The management of NAFLD includes the control of the main cardiometabolic risk factors, the correction of all the modifiable risk factors associated with fibrosis progression, and the prevention of liver-related and extra-hepatic complications [21]. An emerging field of interest has been the relationship between coffee intake and NAFLD (Figure 1). Wadhawan and Anand (2016) have shown that coffee consumption was associated with improved liver enzymes (ALT, AST, and GGT), especially in individuals at risk of liver disease [22]. This improvement was less consistent in patients at higher risk of liver damage [23].

It has also been widely demonstrated that patients with pre-existing liver disease who drank more than three cups of coffee per day had a reduced risk of fibrosis and cirrhosis [24]. Although evidence suggests that coffee intake may reduce the risk of developing NAFLD, data are scarce concerning its role in fibrosis progression in patients with NAFLD [25,26]. However, recent guidelines have included coffee as a non-pharmacological treatment of NAFLD, now termed MASLD, as stated before in this review [26].

Several epidemiological studies have reported that regular coffee consumption was inversely correlated with liver fibrosis [27,28,29,30], indicating that these beneficial effects are not etiology-dependent [25]. Much experimental evidence confirms the role of coffee in preventing liver fibrosis, most of which involves patients with alcohol use disorder, NAFLD, or HCV infection [31,32,33,34]. Coffee drinkers are less likely to develop liver fibrosis and cirrhosis than non-drinkers, as shown by the paired analysis. Importantly, a prospective, multicenter, population-based cross-sectional study in patients with NAFLD showed that high coffee consumption correlated with a clinically significant lower incidence of liver fibrosis (8.8% vs. 16.3%, *p* = 0.038). This study ruled out an association between coffee consumption and new-onset NAFLD, but a multivariate regression analysis evidenced that in patients already presenting steatosis, fibrosis severity was inversely related to coffee consumption [35].

Results from animal models demonstrated that coffee is able to reduce inflammatory cytokines, to modify gene expression in adipose tissue, and to limit collagen deposition in the liver [36,37]. In addition, histological studies showed that coffee consumption counteracts the onset of liver fibrosis [32]. Some studies addressed the molecular mechanisms by which caffeine exerts beneficial effects on the liver, hypothesizing a blockade of the expression of TGF-β and its connective tissue growth factor, with a consequent decrease in the mRNA levels of these profibrogenic proteins. Furthermore, caffeine inhibits hepatic stellate cell activation by blocking smooth muscle actin expression, attenuating inflammatory and fibrotic processes (Table 1) [9,22,38,39]. A reduction in liver stiffness but not in liver fat content was shown in subjects who drank more than three cups of coffee a day, thus suggesting that coffee has an antifibrotic effect since caffeine, an adenosine receptor antagonist, reduces fibrogenesis and thus collagen production [37]. A recent meta-analysis found a significant reduction (35%) in liver fibrosis associated with coffee consumption [40].

### 2.3. Liver Cirrhosis

All causes of chronic liver disease can progress to cirrhosis, characterized by a dynamic and progressive course with a long asymptomatic period of liver inflammation, the remodeling of the extracellular matrix, and collagen deposition in the liver tissue, underlying the development of fibrosis. This process, together with focal hyperplasia and the proliferation of hepatocytes, causes an alteration of the liver architecture and the development of regenerative nodules. The cells involved in this process are HSCs, sinusoidal endothelial cells, and Kupfer cells, which play a role in promoting liver fibrosis. Hepatic stellate cells, in response to a pathogenic insult, are activated by inflammatory cytokines and growth factors, and transform into myofibroblasts, depositing collagen and promoting fibrosis. The sinusoidal endothelial cells undergo remodeling and capillarization, which causes increased resistance in the intrahepatic portal circulation. As the disease progresses, complications such as acute-on-chronic liver failure, hepatic encephalopathy, bacterial infections, hepatorenal syndrome, and HCC may occur [12,41,42,43].

### 2.4. Hepatocarcinoma

Cirrhosis is the principal risk factor for HCC even though it may also occur in the absence of cirrhosis. The risk to develop HCC in non-cirrhotic patients with NAFLD is higher than in patients with liver disease of viral or alcohol-related etiology, as reported by retrospective studies. In an Italian multicenter prospective study, 54% of the patients with NAFLD and HCC were non-cirrhotic [44]. Among the most common risk factors for the development of HCC, in addition to those long known such as HBV and HCV infection, alcohol and tobacco consumption, and age, dietary habits also play an important role. Subjects who consume high amounts of red meat and animal fats show higher levels of the markers of inflammation and endothelial dysfunction correlated with an unfavorable outcome in patients with cirrhosis and HCC [45]. The association between coffee intake and the urinary concentrations of 8-hydroxydeoxyguanosine (8-OHdG), a biomarker of systemic oxidative damage and DNA repair, was analyzed: by examining more than 200 female subjects, the data showed that the urinary concentrations of 8-OHdG tended to decrease in women with a daily intake of 2–3 cups of coffee. Coffee consumption may be associated with a decrease in systemic oxidative DNA damage by decreasing iron accumulation in the body [46]. HCV carriers have a 15- to 20-fold increased risk of developing HCC compared to non-carriers. Moreover, patients with co-infection HBV/human immunodeficiency virus, males, elderly, excessive alcohol drinkers, heavy smokers [9], and patients with visceral obesity have shown an increased risk of developing HCC [47]. Interestingly, serum leptin levels are higher in patients with HCC, indicating the role of this adipokine as a promoter of HCC in obese patients [47]. In contrast, protective nutritional factors include diets rich in fiber, vegetables, fruit, ω-3 polyunsaturated fatty acids, and coffee. Numerous studies indicate that polyphenols including hydroxycinnamic acids (caffeic acid and curcumin), flavanols (quercetin, catechin, and epigallocatechin gallate), flavanones (hesperetin), isoflavones (genistein), anthocyanidins (cyanidin and procyanidin), and stilbenes (resveratrol), mainly in fresh fruit and vegetables, perform their anticarcinogenic mechanism through the upregulation of tumor suppressors and autophagy by modulating some metabolic signaling pathways, thus reducing the risk of HCC [48,49]. The protective role of coffee for the cancer of the digestive tract (oral cavity, esophagus, stomach, and colorectal tract), liver cancer, and liver disease has been well documented to be caused by polyphenols [50,51]. Interestingly, a daily intake of two or more cups of coffee containing caffeine and, to a reduced extent, decaffeinated coffee, was associated with a reduced HCC risk even in the presence of pre-existing liver disease [52]. In a 2015 prospective case–control study, Aleksandrova et al. [53] studied the potential role of inflammatory, metabolic, liver damage, and iron metabolism mediators on the inverse association between HCC risk and coffee consumption, as suggested by the reduction in IL-6 levels, a biomarker of inflammation and innate immunity, together with reduced hepatocellular and cholestatic damage biomarkers, i.e., GLDH (glutamate dehydrogenase), ALT (alanine aminotransferase), AST (aspartate aminotransferase), GGT (γ-glutamyl transferase), and bilirubin. IL-6 is a pleiotropic cytokine produced by the liver during the acute response, active in immune regulation, inflammation, and oncogenesis, and therefore involved in the pathogenesis of liver cancer and its concentration decreases with increasing coffee intake. Furthermore, a dose-dependent improvement in serum enzyme concentrations, especially AST and GGT, was associated with coffee consumption [54]. High coffee intake is also associated with lower concentrations of GLDH, a liver-specific mitochondrial enzyme associated with toxic parenchymal liver damage. Numerous studies have been conducted to identify the relationship between coffee consumption and cancer, especially liver cancer, demonstrating an inverse correlation between coffee consumption and HCC. A meta-analysis published in 2013 reported a 40% reduced risk of developing HCC in patients who consumed coffee [RR(relative risk): 0.60]; the inverse association may also be explained by the fact that patients affected by liver and digestive diseases tend to reduce coffee consumption [55]. High coffee consumption (≥3 cups/day) results in a greater protective effect (RR: 0.44) than lower consumption (<2 cups/day, RR: 0.72). An increase of 1 cup/day correlates with a reduction in RR (RR: 0.80). Drinking 2–3 cups of coffee per day reduces the risk of developing HCC by 38%; a 41% risk reduction (RR: 0.59) was shown in drinkers of ≥4 cups/day. The beneficial effects of coffee are reported for ≥2 cups/day. Increased beneficial effects have been reported for up to 4–6 cups of coffee per day, and one cup corresponds to 10 g whole bean coffee and to 5 g soluble coffee. A caffeine intake of 400 mg/day is considered safe; the 2018 EASL guidelines on HCC [43] recommend coffee consumption in patients with chronic liver disease to reduce the risk of HCC. The studies also show that only American coffee was related to the reduction in liver fibrosis, cirrhosis, and HCC but not Turkish coffee (prepared with boiling coffee grounds without filtration) nor espresso (prepared with pressurized hot water). The reasons are still not entirely clear, but it is speculated that the addition of refined sugar in espresso and Turkish coffee may mask the beneficial effects of coffee. A further explanation could be linked to the different caffeine intake, which is lower in American coffee, but consumed in larger quantities, than in espresso and Turkish coffee [56]. In 2016, the International Agency for Research on Cancer (IARC) evaluated the association between coffee consumption and the risk of liver cancer through a comprehensive review of 14 cohort studies and 11 case–control studies. The findings of the Working Group’s analysis revealed a significant inverse correlation between coffee consumption and the likelihood of developing liver cancer [57].

## 3. Roles of Bioactive Components of Coffee in Liver Disease

### 3.1. Caffeine

#### 3.1.1. Pharmacokinetics

The white, odorless powder known as caffeine (1,3,7-trimethylxanthine) has a bitter taste and is soluble in both water and lipids. It is quickly absorbed from the digestive tract primarily from the stomach and small intestine. Caffeine content in saliva ranges from 65 to 85% of plasma levels. The limited binding to plasma proteins, together with the hydrophobic properties of caffeine, allow its passage through all the biological membranes, including the blood–brain barrier and the placenta. After oral administration, caffeine reaches systemic circulation in a few minutes. The absolute bioavailability of caffeine is very high and reaches almost 100% [58,59]. Its absorption does not depend on age, sex, genetics, disease, or on the consumption of drugs, alcohol, or nicotine.

The Tmax after oral doses between 72 and 375 mg of caffeine in healthy adult volunteers varies between 15 and 60 min, and the peak plasma concentration (Cmax) after the oral dosing of caffeine 70–500 mg is variable (5–50 µM [59]. The degree of caffeine degradation appears to differ between individuals due to both environmental and genetic influences [60]. The hepatic cytochrome P450 1A2 (CYP1A2) is part of the mixed-function oxidase system responsible for metabolizing and detoxifying xenobiotics in the body. It is responsible for metabolizing > 95% of the caffeine in the liver [61]. CYP1A2 is responsible for the process of demethylating caffeine, resulting in the production of the main metabolites [62] paraxanthine (1,7-dimethylxanthine, around 84%), theobromine (3,7-dimethylxanthine, 12%), and theophylline (1,3-dimethylxanthine, 4%). Additionally, less than 6% of the process involves C-8 hydroxylation, producing 1,3,7-trimethyluric acid. Three to five percent of these three caffeine metabolites remain as caffeine when eliminated through urine after undergoing additional demethylation and oxidation in the liver. Paraxanthine and its derivatives [1, 7-dimethyluric acid, 1-methyluric acid, 1-methylxanthine, and 6-amino-5-(N-formylmethylamino)-3-methyluracil] are the primary urine metabolites in humans [63].

The half-life of caffeine varies between 2.5 and 5.0 h in adults [59,64,65], and it depends on age, sex, and the use of certain drugs such as oral contraceptives, carbamazepine, rifampicin, cimetidine, or ciprofloxacin. Caffeine can also cross the placenta and may be present in breast milk. Furthermore, some conditions, such as pregnancy, smoking, and liver disease, are associated with the increase in the half-life of caffeine [66]. High intake of caffeine causes the saturation of its metabolism [59]. Thus, linear pharmacokinetics were observed for caffeine intake between 70 and 100 mg; in contrast, 250–500 mg of caffeine caused a significant increase in its plasma concentrations, with non-linear kinetics and prolonged half-life.

A healthy adult should not take more than 400 mg of caffeine per day, not exceeding 3 mg/kg/day of caffeine in a single dose, according to the European Food Safety Authority (EFSA). Pregnant or nursing women should limit their daily intake to <200 mg [67,68,69]. The excretion of caffeine is renal. The plasma concentrations of caffeine decrease more rapidly than its metabolite paraxanthine within 8–10 h of administration [59,65].

#### 3.1.2. Pharmacodynamics

When caffeine is absorbed, it acts on both the central and peripheral nervous system [68,69,70,71]. Caffeine’s action is due to its ability to antagonize adenosine receptors that are widely present in all the organs and tissues of the human body [56].

The inhibition of phosphodiesterase, the release of calcium from intracellular reserves, the antagonistic action on benzodiazepine receptors, and the antioxidant properties are thought to be additional mechanisms by which caffeine exerts its activity [70,71,72]. Caffeine’s capacity to antagonize adenosine receptors plays a role in its impact on behavior and cognitive function, thus resulting in its central nervous system effects, especially those related to adenosine’s neuromodulatory properties. The release of adrenaline, dopamine, acetylcholine, serotonin, glutamate, gamma-aminobutyric acid (GABA), and possibly neuropeptides is indirectly influenced by caffeine, as it prevents the inhibitory effects of adenosine through its receptors [72]. There are four adenosine receptors: A1, A2A, A2B, and A3; caffeine is a non-selective antagonist of these receptors. Adenosine is an endogenous inhibitory neuromodulator that causes feelings of drowsiness, and therefore, caffeine generally induces stimulatory effects in the central nervous system. Its physiological effects also include an acute increase in blood pressure, metabolic rate, and diuresis [73].

Circulating plasma levels of caffeine (10–50 µM) selectively block A1 and A2A receptors and competitively inhibit the action of adenosine, while higher concentrations are necessary to inhibit A2B and A3 subtypes [59,74,75]. It was found that caffeine exerts antifibrotic activity by inhibiting the activation of the A2A receptor [76] through the upregulation of peroxisome proliferator-activated receptor γ (PPARγ) and reduction in SMAD2/3 [77]. In addition, caffeine inhibited matrix metalloproteinase (MMP) secretion and reduced α-smooth muscle actin (α-SMA) in liver fibrosis (Table 1) [78]. HSCs express adenosine A1 and A2A receptors, and acetaldehyde activates these receptors, causing HSCs to become activated and proliferate. Furthermore, in alcoholic liver fibrosis, adenosine A1A and A2A antagonists as well as caffeine have a strong inhibitory effect on HSC activation and proliferation [79]. Caffeine has been found to stimulate the release of norepinephrine, dopamine, and serotonin, leading to improvements in psychomotor properties and behavioral functions such as mood, well-being, energy levels, alertness, mental concentration, attention, and memory [72,80,81]. Caffeine inhibits phosphodiesterase enzymes in muscle, which results in increased intracellular cyclic adenosine monophosphate (cAMP) concentrations and activates hormone-sensitive lipases in skeletal and adipose tissues [72]. As a result, this promotes the release of free fatty acids and glycerol by stimulating lipolysis. There are 11 different families of phosphodiesterases (PDEI-PDEII) that have slightly different functions and tissue specificities [81]. Within each family of phosphodiesterases, several subtypes may exist that exert various effects depending on their activation and inhibition. Depending on the inhibited isotype, the pharmacological inhibition of tissue-specific phosphodiesterases can have a variety of therapeutic effects [81]. However, caffeine is a weak inhibitor of phosphodiesterases and the in vivo concentrations at which behavioral effects occur are probably too low to be associated with significant phosphodiesterase inhibition [82,83]. In contrast, phosphodiesterase inhibition may explain the cardiostimulatory and antiasthmatic actions of caffeine and theophylline [84] and are also effective as bronchiolar and tracheal relaxants [85,86]. For this reason, caffeine is used as a pharmacological treatment in neonatal intensive care in the management of the complications of apnea in premature newborns; in fact, it reduces the duration of ventilation and dependence on oxygen and improves disability and survival [87]. The mobilization of intracellular calcium (Ca^2+^) is also caused by caffeine [88]: on the one hand, it inhibits the second messenger inositol triphosphate (IP_3_) decreasing the release of calcium from the endoplasmic reticulum; on the other hand, however, it activates the ryanodine receptors (RyR) which play a key role in the excitation–contraction processes of the skeletal muscle, thus contributing to the release of calcium from the rough endoplasmic reticulum and, in turn, to the entry of calcium into circulation from the extracellular space. This is a key event in T-cell activation and cytokine production. The modulation of intracellular Ca^2+^ homeostasis and excitation coupling is a critical function of RyRs, making them prospective therapeutic targets for different disorders, including neurodegeneration, myopathies, malignant hyperthermia, and cardiovascular [89].

At high concentrations of 1–10 mM, caffeine disrupts the uptake and retention of Ca^2+^ in the sarcoplasmic reticulum of striated muscle and enhances the movement of Ca^2+^ through the cell membrane [71]. The threshold concentration of caffeine (250 µM) used in vitro to release calcium from intracellular (sarcoplasmic reticulum) stores in skeletal and cardiac muscle is higher than the concentrations required in vivo for cardiac stimulation (50 µM) [90]. The increased calcium level in the hepatic endoplasmic reticulum (ER) inhibits the transcriptional activation of sterol regulatory element-binding protein 2 (SREBP2). This protein is responsible for regulating PCSK9 expression, leading to an increased expression of LDLR and promoting the clearance of low-density lipoprotein cholesterol (LDLc) [91]. This supports the idea that coffee consumption has a beneficial effect on lipid metabolism disorders, resulting in a cholesterol-lowering effect.

Caffeine is a weak antagonist of benzodiazepine receptors, i.e., the GABA (gamma amino butyric acid, an inhibitory amino acid) receptor, thus leading to increased attention, anxiety, and seizure activity [92,93,94]. The chronic exposure of embryonic brain neurons to caffeine or theophylline reduces the ability of GABA to enhance the binding of [^3^H]flunitrazepam to the GABA/benzodiazepine receptor. Adenosine blocks the theophylline-induced “uncoupling” of the allosteric interactions of GABA and benzodiazepine binding sites, suggesting that the long-term effects of theophylline are caused by an adenosine receptor. However, this mechanism requires very high concentrations [95,96]. Some of the beneficial effects reported for caffeine have been associated with its antioxidant actions, although some studies have not observed this activity at normal concentrations [97]. It has been highlighted that caffeine is an effective scavenger of hydroxyl radicals generated by the Fenton reaction. In addition, some caffeine metabolites, such as 1-methylxanthine, 1-methyluric acid, and 1-methylurate, have antioxidant properties. For instance, the antioxidant activity of 1-methylxanthine is comparable to that of ascorbic acid. Caffeine’s anti-inflammatory effects are likely due to its ability to inhibit phosphodiesterase and the antagonism of adenosine receptors. Caffeine has been shown to increase the release of anti-inflammatory cytokines, such as interleukin-10 (IL-10). Moreover, caffeine mitigates immune suppression by releasing pro-inflammatory cytokines such as interferon gamma (IFN-γ), IL-2, and tumor necrosis factor alpha (TNF-α), which are crucial for the development and progression of autoimmune disorders [98].

### 3.2. Chlorogenic Acids

Plants contain large groups of phenolic acid molecules, known as chlorogenic acids (CGAs), which are a family of quinic acid esters coupled with C6–C3 trans-hydroxycinnamic acid. The primary members of this family include ferulic acid (FA), caffeic acid (CA), and p-coumaric acid. CGAs are found in many different beverages including tea and coffee. The main CGAs in coffee is 5-O-caffeoylquinic acid (5-CQA) (Figure 1). A 200 mL cup of coffee may contain a minimum of 20 to a maximum of 675 mg of CGAs; its amount may vary depending on the coffee type and the method of preparation [99].

#### 3.2.1. Pharmacokinetics

Pharmacokinetics studies in humans have demonstrated that CGAs are absorbed to a limited extent in the upper digestive tract and are metabolized quickly; the low absorption and bioavailability of CGAs are due to their hydrophilic nature. It is mainly absorbed in the intestine through passive diffusion; in the intestine, it undergoes partial hydrolysis, and then, sulfation and glucuronidation are implied in its metabolism. The unabsorbed portion of CGAs is extensively hydrolyzed by the gut [100].

After coffee consumption, Cmax is found at 0.5 to 1.0 h. However, the human metabolism of CGAs is somewhat complex but well defined [101]. One-third of CGAs (as 5-CQA) are adsorbed intact in the stomach or upper intestine and then pass into the bloodstream. The other two-thirds pass into the colon and interact with the intestinal microflora, and then are excreted with feces [102]. After consuming coffee, the primary CGAs-related compounds are caffeic acid-3′-O-sulfate and ferulic acid-4′-O-sulfate, which are absorbed in the small intestine and briefly appear in the bloodstream after 1 h (Tmax).

#### 3.2.2. Pharmacodynamics

CGAs can reduce the NF-κB, JAK, and MAPK pathways by blocking the production of pro-inflammatory mediators including TNF-α, NO, COX-2 expression and PGE_2_ biosynthesis, and IL. By inhibiting inflammatory factors, slowing the spread of inflammation, and avoiding tissue damage caused by inflammation, CGAs can combat the components of the inflammatory pathway on several levels.

They can affect the inflammatory pathway on several levels, reducing the release of pro-inflammatory factors and thus counteracting the inflammatory process and its associated tissue damage [101,103]. In addition, CGAs cause the increase in antioxidant factors such as HO-1 and NOQ-1 and the removal of cellular free radicals [104]. Additionally, they can modulate glucose release and uptake, lipid synthesis, and the AMPK pathway, all of which assist to regulate and maintain lipid and glucose metabolic balance. [101]. Finally, CGAs exhibit neuromodulation by targeting multiple neuroreceptors and channels such as GABA receptors, potassium channels, and acid-sensitive ion channels, achieving antinociceptive effects [105]. By blocking the Smad7 pathway, which is controlled by miR-21, TGF-β1, or IL-13, CGAs prevent liver fibrosis [106]. By inhibiting oxidative stress [107] and activating HSCs, CGAs protect against CCL4-induced liver fibrosis both in vitro and in vivo by producing vascular endothelial growth factor (VEGF) and TGF-β1. Additionally, CGAs decrease fibrosis and inflammation by suppressing the Toll-like receptor 4 (TLR-4) pathways [108].

CGAs also exert protective actions on fibrosis in non-alcoholic steatohepatitis by downregulating multiple pro-fibrogenic factors and oxidative stress via the HIF-α/miR-122 and Nrf2 pathways, respectively [109]. Thus, CGAs prevent oxidative stress, inflammation, and fibrosis in HSCs and fibroblasts by inhibiting the miR-21/Smad7/TGF-β1/IL-13/TLR-4/HIF-α/miR-122 and Nrf2 signaling pathways. The protective role mediated by CGAs could also be involved in pulmonary, renal, and cardiac fibrosis.

Several experimental pieces of evidence have demonstrated the anti-cancer effect of CGAs [110]: importantly, their supplementation potentiates regorafenib (a multikinase inhibitor targeting the RAS/RAF/MEK/ERK pathway) activity in human HCC [111] and promotes the effect of 5-fluorouracil in HCC cells by reducing (i) the extracellular signal-regulated kinases (ERKs) (in vitro and in vivo) and (ii) DNMT1 expression [105]. Furthermore, CGAs enhance the apoptosis mediated by oxidative stress in hepatocytes [112,113,114,115].

## 4. Conclusions

Coffee is one of the most largely consumed beverages in the world and contains several bioactive compounds with potential effects on human health, including caffeine, CGAs, kahweol, diterpenes, cafestol, polyphenols, and melanoidins. The consumption of this beverage has long been controversial in the scientific community. Although its consumption is advised against in specific clinical settings, more encouraging research suggests that it may have protective and beneficial effects against a variety of chronic metabolic diseases, including diabetes, cardiovascular diseases, and neurodegenerative illnesses such as Parkinson’s and Alzheimer’s disease [6,7,8,9,10]. In recent decades, moreover, epidemiological and experimental studies have shown the inverse correlation of coffee consumption with the incidence and progression of chronic liver diseases such as NASH, viral hepatitis, liver cirrhosis, HCC, and liver-related mortality [9,27,28,29,30,31,32,33,34,35]. Its consumption is inversely associated with the activity of liver enzymes, GGT, and alanine aminotransferase in at-risk individuals including alcohol drinkers, and promotes an improvement in hepatic steatosis and fibrosis, and a lower risk of developing cirrhosis and HCC. The protective effects are independent of the etiology of liver disease.

The results of a meta-analysis showed a decreased risk (29–30%) of NAFLD and liver fibrosis in regular coffee drinkers [34]. Interestingly, a retrospective study found that high coffee consumption (>3 cups/day) was associated with lower hepatic fibrosis (50%) [35]. Moreover, a lower risk of liver cancer was found in HBV patients drinking coffee ≥4 times per week [17]. In addition, it was found that coffee consumption was inversely related to death from chronic liver disease and HCC as shown by prospective observational studies [26,116]. Prospective observational studies have indicated a link between the consumption of various types of coffee (decaffeinated, instant, and ground coffee) and protection against chronic liver disease and HCC [26,116]. Recently, the associations of the consumption of different coffee types (decaffeinated, instant, and ground coffee) with chronic liver disease were assessed in a large prospective cohort of 494,585 UK Biobank participants with a median follow-up of 10.7 years: significant protection against chronic liver disease was found for all types of coffee without any difference [116].

The main beneficial effects related to coffee consumption are antioxidant, antifibrotic, insulin-sensitizing, and anticarcinogenic. There is evidence of a modulation of the gene expression of the enzymes involved in fatty acid synthesis and hepatic collagen production, a reduction in oxidative stress with an increase in glutathione, and a decrease in pro-inflammatory cytokines. Although epidemiological results show that coffee consumption is inversely correlated with liver cirrhosis, a causal preventive role of liver damage cannot be demonstrated. The inverse correlation is dose-dependent; in fact, coffee consumption ≥2 cups/day protects against the progression of almost all forms of liver disease with incremental beneficial effects up to 6 cups/day. Up to 400 mg of caffeine per day is considered safe. Young individuals who consume excessive amounts of coffee need to be cautioned about possible side effects like headaches and insomnia, as well as the potential danger of caffeine withdrawal [117]. Among the bioactive substances present in coffee, the role of phenolic compounds of natural origin, CGAs, which appear to show multiple pharmacological effects, is increasingly studied. The suppression of inflammation, cellular senescence, the modification of the extracellular matrix, the TGF-β overproduction, and fibroblast proliferation and differentiation are responsible for the antifibrotic role of CGAs in the liver (Table 1) [97,98,99,100,101,102,103,104,105]. However, the specific molecular mechanism remains unclear [118]. Few studies evaluated the effect of the combination of CGAs and caffeine on liver disease. The study by Zheng et al. (2014) showed that the interaction between caffeine and CGAs impacts the functions of the enzymes related to lipid metabolism by controlling their mRNA and protein expression. The increase in fatty acid oxidation and the suppression of fatty acid synthase activity were responsible for the reduced serum and hepatic lipid levels in mice [119].

In addition, interestingly, a recent in silico study has confirmed the positive beneficial effect of caffeine on liver cirrhosis [120].

In conclusion, coffee, even if with many beneficial effects, is still considered a beverage and not a drug [26] for the primary and secondary prevention of chronic liver disease. The data in favor of coffee consumption in patients with liver disease are encouraging, but we still need prospective studies to demonstrate its preventive and therapeutic role in chronic liver diseases. While waiting for new scientific evidence and more clarification, we can affirm that a daily moderate use of coffee (without sugar) is a reasonable non-pharmacological treatment for these patients, always keeping in mind the great importance of adopting a correct lifestyle, not exceeding fat and alcohol intake, performing constant physical activity, and regular screening. Coffee should, therefore, be recommended in association with correct lifestyle habits, although further prospective studies should be performed to endorse its protective effect.

## Figures and Tables

**Table 1:** Antifibrotic activity of caffeine and CGAs.

Component of Coffee	Mechanism
Caffeine	The upregulation of PPARγ and reduction in SMAD2/3 The inhibition of α-smooth muscle actin expression The enhancement of HSC apoptosis and intracellular F-actin and cAMP expression The inhibition of procollagen type 1C expression
CGAs	The suppression of inflammation The modification of the extracellular matrix, The increased biosynthesis of TGF-β Fibroblast proliferation and differentiation

## Data Availability

The original contributions presented in the study are included in the article, further inquiries can be directed to the corresponding author.
